# Specificity of Arsenic Stress Detection by Raman Spectroscopy During Co-Occurrences of Nitrogen Deficiency and Narrow Brown Leaf Spot

**DOI:** 10.1021/acs.analchem.5c05673

**Published:** 2025-12-18

**Authors:** Isaac D. Juárez, Myles Russwurm, Sabin Khanal, Sudip Biswas, Endang M. Septiningsih, Xin-Gen Shane Zhou, Dmitry Kurouski

**Affiliations:** † Department of Biochemistry and Biophysics, 14736 Texas A&M University, College Station, Texas 77843, United States; ‡ Interdisciplinary Faculty of Toxicology, Texas A&M University, College Station, Texas 77843, United States; § Texas A&M AgriLife Research Center, Beaumont, Texas 77713, United States; ∥ Department of Soil and Crop Sciences, Texas A&M University, College Station, Texas 77843, United States

## Abstract

Arsenic contamination in rice poses a potential health risk to populations dependent on their daily consumption. Previous work has shown that Raman spectroscopy is capable of nondestructively diagnosing arsenic uptake in rice; however, its diagnostic specificity in cases of concurrent abiotic and biotic stress remains unclear. As Raman spectroscopy relies on the detection of arsenic-induced stress patterns for diagnosis, the presence of additional stressors could potentially compromise diagnostic reliability. To address this gap, we evaluated the ability of Raman spectroscopy to detect arsenic uptake in the presence of both nitrogen deficiency (abiotic stress) and narrow brown leaf spot (biotic stress) across two Experiments. We found that nitrogen deficiency, while more severe than arsenic stress, did not affect arsenic detection. We also found that the diagnostic accuracy for both abiotic stressors (arsenic and nitrogen deficiency) depended on the plant growth stage, with arsenic detection being most reliable immediately after transplantation and nitrogen deficiency becoming more distinguishable as stress severity increased. Narrow brown leaf spot, though exhibiting minimal symptoms, remained sufficiently detectable. Altogether, these findings demonstrate that Raman spectroscopy remains a reliable method for diagnosing arsenic uptake and assessing overall rice health, even in the presence of additional stressors.

## Graphical abstract

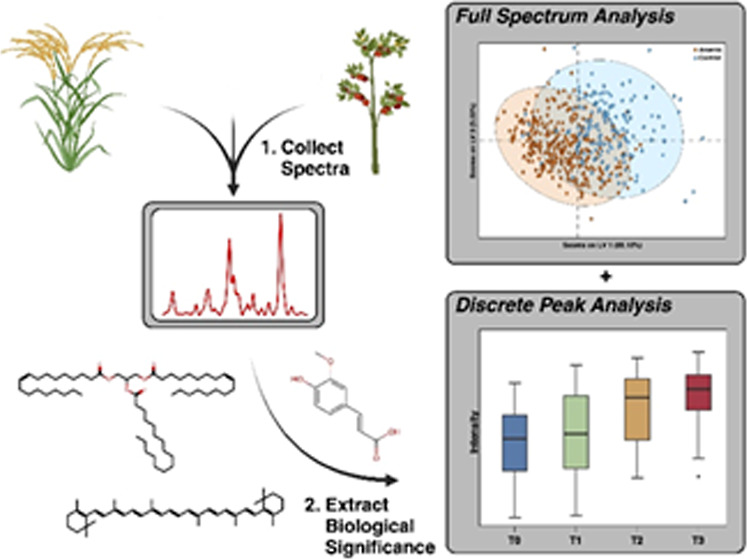

## Introduction

Arsenic contamination within the environment is ubiquitous, arising from both natural and anthropogenic sources.^1–4^ Areas with high contamination frequently coincide with major rice production regions.^5,6^ Combined with rice’s strong tendency to bioaccumulate arsenic, rice and its derived products have become the major dietary source of inorganic arsenic exposure in humans.^7–9^ Conventional detection methods, such as ICP-MS and related quantitative approaches,^10–13^ provide high precision and low limits of detection. However, their destructive nature and technical complexity limit their application for routine crop monitoring and proactive management.

Raman spectroscopy (RS), a technique based on the inelastic scattering of light, has found a diverse usage in agricultural research as a promising nondestructive diagnostic sensor.^14^ It has been shown that RS could be used for highly accurate diagnostics of fungal, viral, and bacterial diseases in plants.^15–23^ For instance, RS was capable of detecting and identifying Huanglongbing, a bacterial-induced disease of citrus tress.^20^ Furthermore, Sanchez and co-workers demonstrated that RS was capable of differentiating between Huanglongbing and nitrogen deficiency in both orange and grapefruit plants.^20^ RS could be also used to diagnose abiotic stresses in plants.^24,25^ For instance, Morey and co-workers demonstrated that RS could be used to diagnose drought and salinity stresses in peanuts,^22^ while Juárez and co-workers showed that RS could detect heavy metal bioaccumulation in hydroponically grown rice with around 85% accuracy, even at environmentally relevant contamination levels.^26,27^

However, these studies were conducted under controlled growth chamber conditions, which do not fully capture the complexity of field environments. In contrast, rice cultivated in the field is exposed to a wide range of abiotic and biotic stressors. For example, nitrogen deficiency (ND) is a major factor limiting rice productivity, particularly under conditions of inadequate fertilizer application or low nitrogen use efficiency.^28^ On the other hand, diseases such as narrow brown leaf spot (NBLS), caused by the fungal pathogen *Cercospora janseana*, can lead to significant losses in crop yield, reaching up to 40% in epidemic years.^29,30^ Because RS does not directly detect the heavy metal but rather the stress-induced biochemical response, it remains unclear whether arsenic uptake and these other stressors can still be reliably diagnosed when they occur together, even though previous studies have demonstrated RS’s ability to diagnose these stressors individually.^31–33^

To address this knowledge gap, we evaluated the ability of RS to diagnose arsenic uptake, ND, and NBLS, both individually and in combination. Two Experiments were conducted: the first assessed arsenic uptake and nitrogen deficiency, while the second added NBLS infection. Spectral data were analyzed using ANOVA and PLS-DA, with 2D correlation spectroscopy (2D-COS) and peak deconvolution employed to assess the underlying biochemical changes.

## Materials and Methods

### Experiment 1

The experiment was conducted in a greenhouse using rice plants grown in soil-filled pots placed in water-filled plastic bins. Seeds were sown directly, and after 31 days, the most vigorous seedlings were transplanted to one plant per pot. Twenty-eight plants of each of cultivars XP753 and Trinity were grown. The plants received nutrients via an *A*/*B* tank setup, consisting of the following: tank *A* (71 mg/L Ca­(NO_3_)_2_, 42.2 mg/L KNO_3_, 12.375 mg/L Fe-EDDHA, 0.178 mg/L Zn-EDTA, 7.125 μg/L Cu-EDTA, 1.928 Mn-EDTA mg/L, 12.5 μg/L Na_2_MoO_4_) and tank *B* (43.875 KH_2_PO_4_ mg/L, 87.75 (NH_4_)_2_SO_4_ mg/L, 86.625 MgSO_4_ mg/L, 10.875 K_2_SO_4_ mg/L). Water and nutrients were applied every 3 days.

Greenhouse conditions were maintained at ∼12 h day/night cycles, 55% relative humidity, and 29 °C:26 °C day/night temperatures. Arsenic stress and ND conditions were initiated at transplantation. Arsenic was added directly after the tank solutions were administered, maintaining a water concentration of 50 μg/L. ND was modeled by using a modified tank *B* solution lacking (NH_4_)_2_SO_4_. The plants in this experiment were divided into four treatment groups based on the following combinations: control (untreated, nitrogen-complete), As (arsenic-treated, nitrogen-complete), N^–^ (untreated, nitrogen-deficient), and N^–^As (arsenic-treated, nitrogen-deficient) [Figure 1]. The experiment ran for approximately 16 weeks in total, or 10 weeks after transplantation, after which all above-ground plant tissue was harvested.

**Figure 1:**
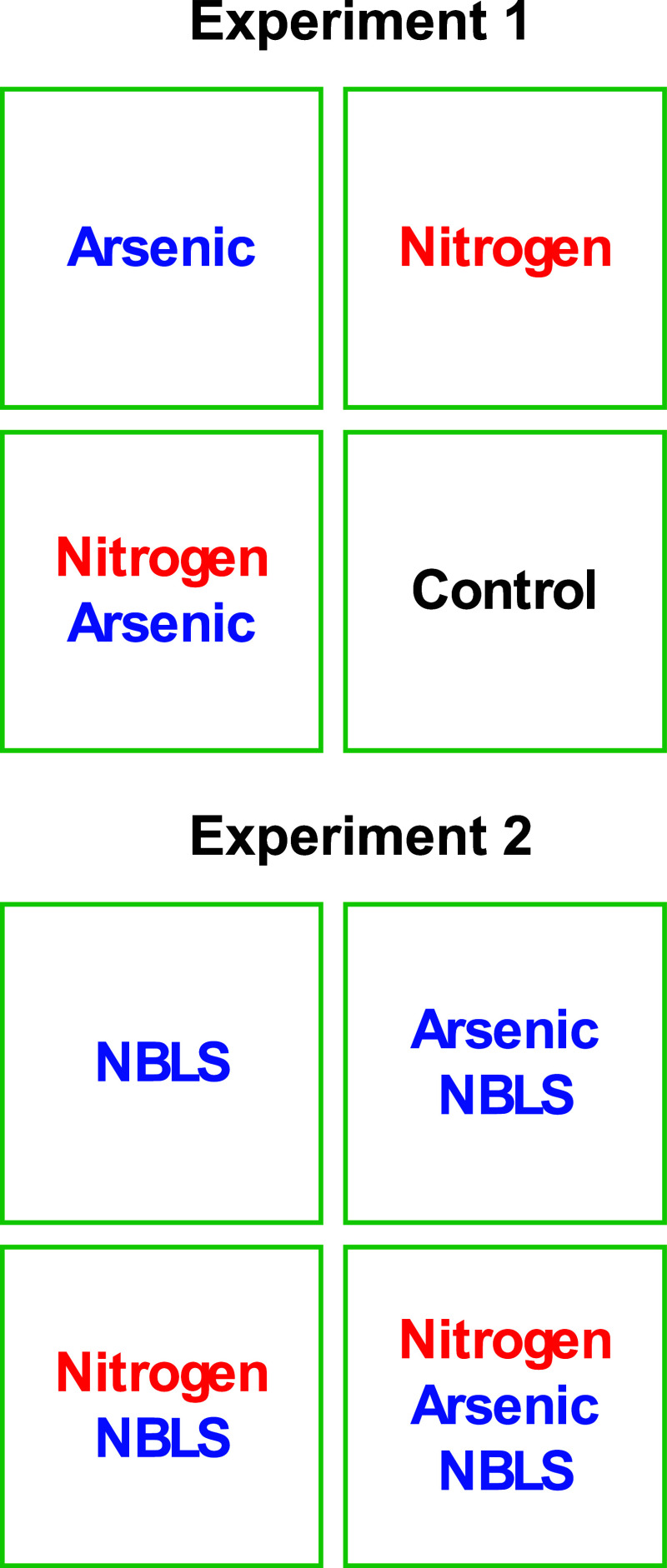
Schematic of experimental conditions used in the study. Experiment 1 (top) evaluated (i) nitrogen depleted, (ii) arsenic intoxicated, (iii) nitrogen depleted with arsenic intoxicated stress, as well as a (iv) control. Experiment 2 includes (i) NBLS infected, (ii) NBLS infected with arsenic stress, (iii) NBLS infected with nitrogen depleted, and (iv) nitrogen depleted with arsenic and NBLS.

For data collection, a minimum of 48 Raman spectra were acquired for each group of plants once a week, and the study was terminated 10 weeks after transplantation. All spectra were baselined and normalized at the 1440 cm^–1^ peak. Plant heights were recorded every 2 weeks, and photographs were taken at the same intervals. Microsoft Excel, R (programming language), and the PLS_toolbox (eigenvector Research Inc.) in MATLAB were used to perform all statistical analyses and construct figures. Data was downloaded from the instrument as CSV files, then imported into each software. ANOVA was performed in R for all peaks with a visual change. Lastly, PLS-DA models were built for a binary comparison of each experimental group, with 4 to 10 LVs used for each model. Temporal graphs were constructed in Microsoft Excel.

### Experiment 2

The experiment was conducted in a greenhouse. Rice plants were cultivated in soil-filled pots placed within water-filled plastic bins. League-type clay soil (3.2% sand, 64.4% silt, 4.3% organic matter, and pH 5.5) was used. Seeds were planted directly into the soil, and after 14 days, the most vigorous seedlings were transplanted, resulting in three plants per pot. A total of 9 plants were used for each treatment in the experiment. The plants received nutrients via the *A*/*B* tank solutions described previously.

Greenhouse conditions were maintained at ∼12 h day/night cycles, ∼60% relative humidity, and 26 °C:16 °C day/night temperatures. The abiotic stress conditions were administered using the procedure described in Experiment 1. The plants were inoculated with the fungal spores of *C. janseana* (the NBLS causing agent) in the fifth week after transplantation. The plants in this experiment were divided into eight treatment groups based on the following combinations: control (untreated, nitrogen-complete, uninoculated), NBLS (untreated, nitrogen-complete, inoculated), As (arsenic-treated, nitrogen-complete, uninoculated), AsNBLS (arsenic-treated, nitrogen-complete, inoculated), N^–^ (untreated, nitrogen-deficient, uninoculated), N^–^NLBS (untreated, nitrogen-deficient, inoculated), N^–^As (arsenic-treated, nitrogen-deficient, uninoculated), and N^–^AsNBLS (arsenic-treated, nitrogen-deficient, inoculated) [Figure 1]. The experiment ran for approximately 12 weeks in total, or 10 weeks after transplantation, after which all above-ground plant tissue was harvested.

At least of 30 Raman spectra were acquired for each treatment group of plants, collected at weeks 3, 5, and 10. Plant heights and photographs were also collected at these time points. Data were processed and analyzed using the same software as in Experiment 1.

## Results and Discussion

In Experiment 1, at 10 weeks after transplantation, arsenic stress had not caused any overt phenotypic differences between arsenic-treated and untreated rice [Figure 2]. In comparison, ND caused mild chlorosis and a reduction in tiller count. The average plant height was statistically unaffected by arsenic stress; however, both ND groups were significantly shorter than nitrogen-complete groups with an approximately 11% difference in height [Figure 3]. In Experiment 2, arsenic stress elicited an overt reduction in tiller number by 10 weeks after transplantation [Figure 4]. ND resulted in a complete lack of tiller production. Chlorosis was evident only in the N^–^ and N^–^NBLS groups but not in the N^–^As and N^–^AsNBLS groups. However, the latter groups only had one to two surviving leaves, roughly half the number of leaves observed in the N^–^ and N^–^NBLS groups. NBLS inoculation altogether produced minimal symptoms, limited to a few small necrotic lesions per plant. As in Experiment 1, plant height was unaffected by arsenic exposure; however, ND significantly reduced height by about 38% compared to nitrogen-complete groups [Figure 3]. NBLS also seemed to suppress growth; however, the difference in height was only statistically significant between the N^–^ and N^–^NBLS groups. Nevertheless, the rice plants in Experiment 2 were less developed overall than those in Experiment 1. By week 10, the rice had not yet reached the reproductive stage, likely due to low temperatures during their growth stages.

**Figure 2:**
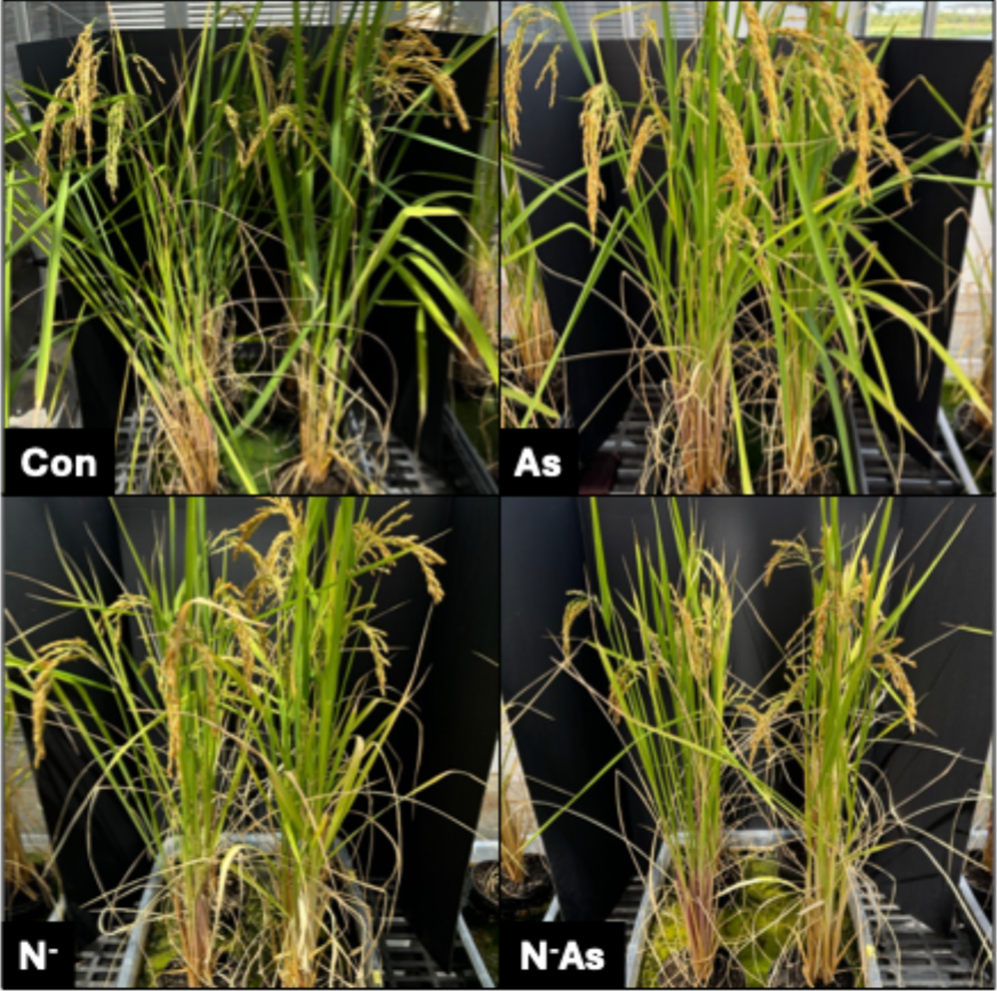
Rice plants at 10 weeks after transplantation in Experiment 1. Con = control, As = arsenic stress, N^–^ = nitrogen deficiency, N^–^As = combined stress.

**Figure 3:**
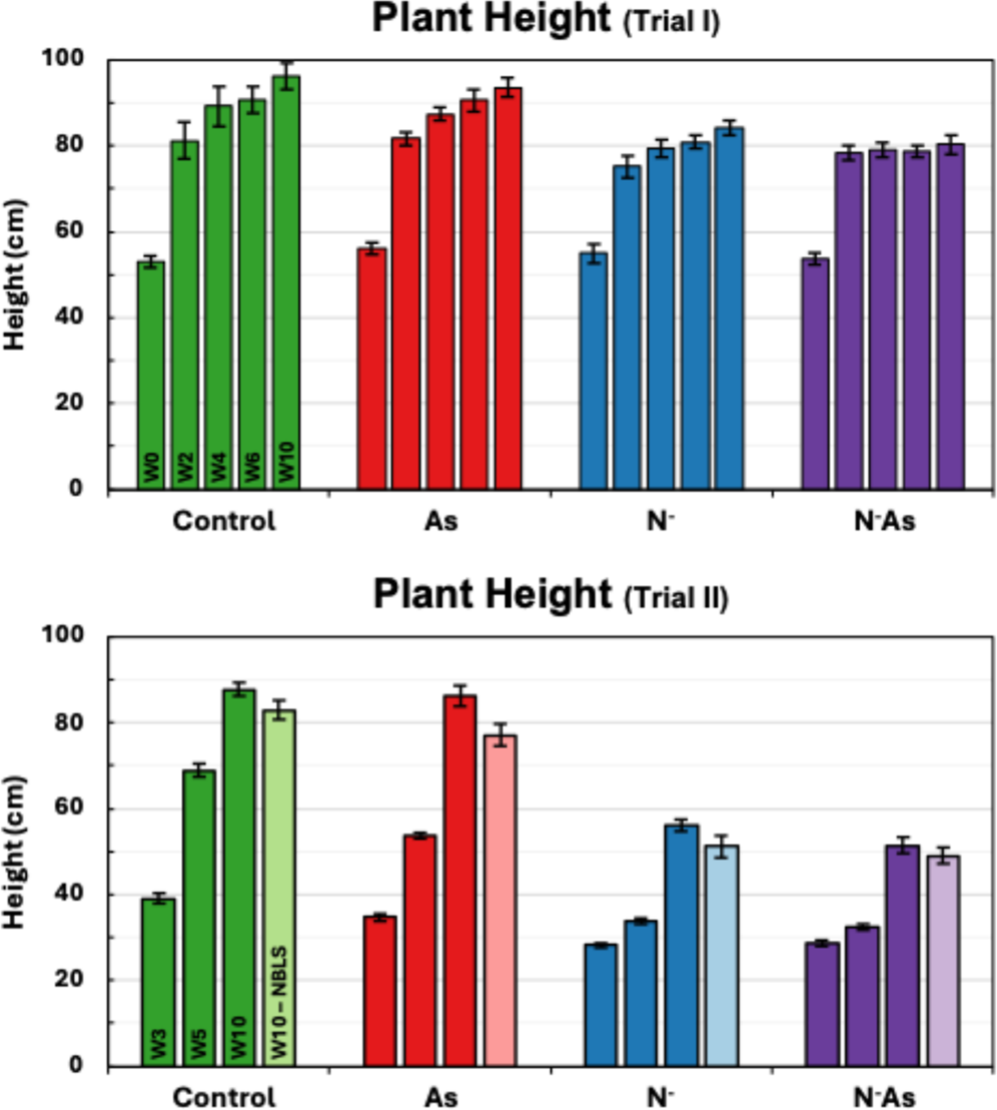
Average rice plant heights by experimental condition in Experiments 1 and 2. Vertical lines indicate the standard error of the mean. As = arsenic stress, N^–^ = nitrogen deficiency, N^–^As = combined stress.

**Figure 4:**
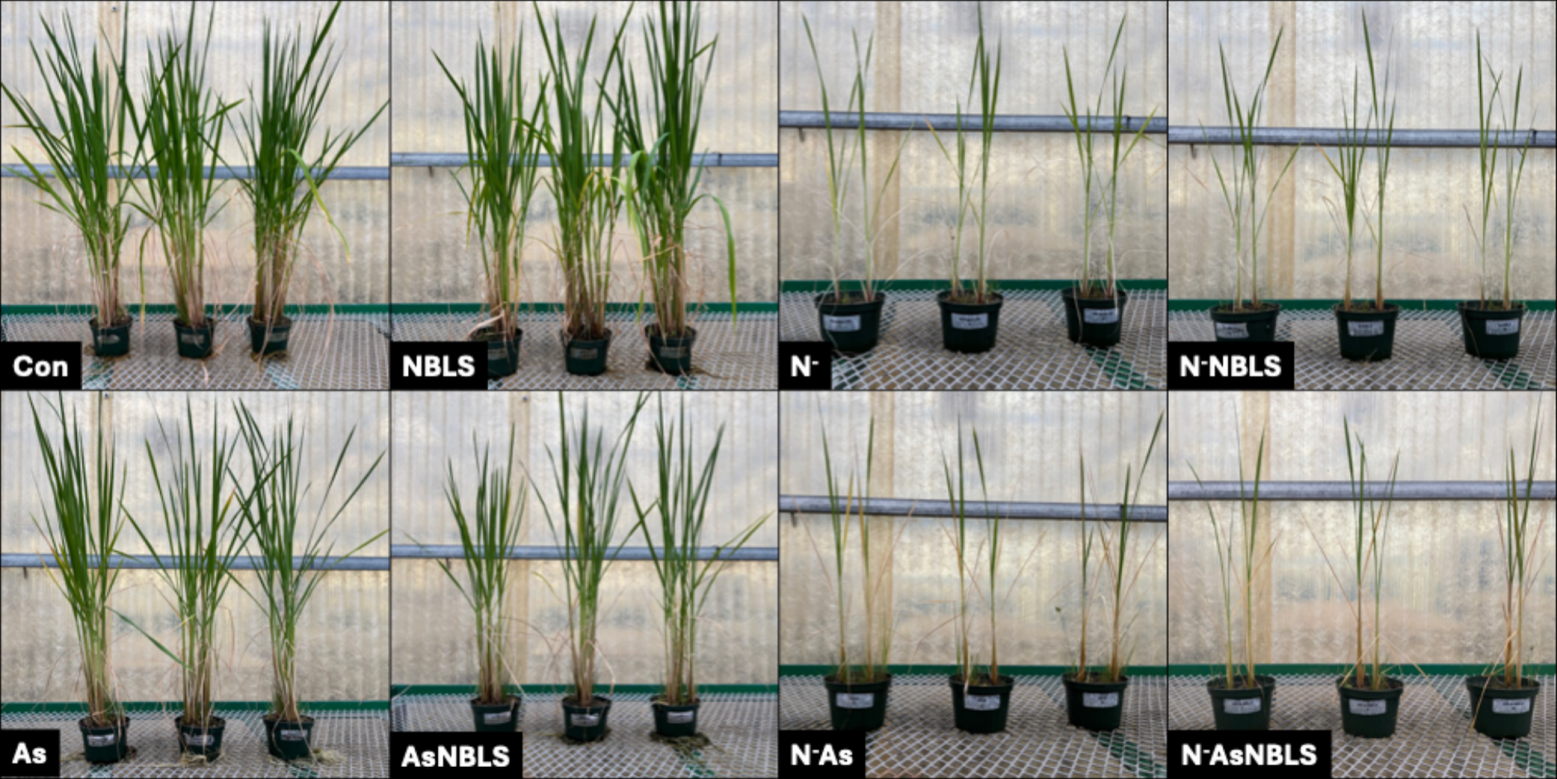
Rice plants at 10 weeks after transplantation in Experiment 2. Con = control, As = arsenic stress, N^–^ = nitrogen deficiency, NBLS = narrow brown leaf spot. All groups indicate a combination of stressors. Pots are 12 cm in width and 16 cm in height.

In Raman spectra collected from the first Experiment, 1 week after transplantation, only carotenoid-related peaks (1001, 1156, 1185, 1216, and 1525 cm^–1^) showed significant differences in intensity [Figure 5A, Table 1 and Figure S1]. By week 10, however, multiple peaks exhibited substantial intensity differences, reflecting alteration in biomolecular content [Figure 5B]. These were primarily peaks associated with amino acids (747, 915 cm^–1^), cellulose (1115 cm^–1^), phenylpropanoids (1601, 1630 cm^–1^), and carotenoids. At week 1, arsenic stress produced the most prominent spectral changes, resulting in significantly higher carotenoid peak intensities, specifically at 1156, 1185, and 1525 cm^–1^, compared to untreated rice. Spectral changes elsewhere were inconsistent or not statistically significant. By week 10, ND groups exhibited significantly lower intensities at carotenoid, cellulose, and amino acid peaks relative to the control. The N^–^As group showed significantly lower carotenoid peak intensities than both the individual As and N^–^ groups, along with a significantly higher phenylpropanoid peak at 1630 cm^–1^. Conversely, the average spectrum of the As group was lower than that of the control at most peaks; however, these differences were not statistically significant. All three stress groups had significantly elevated phenylpropanoid content at the 1601 cm^–1^ peak at week 10 relative to the control.

**Figure 5:**
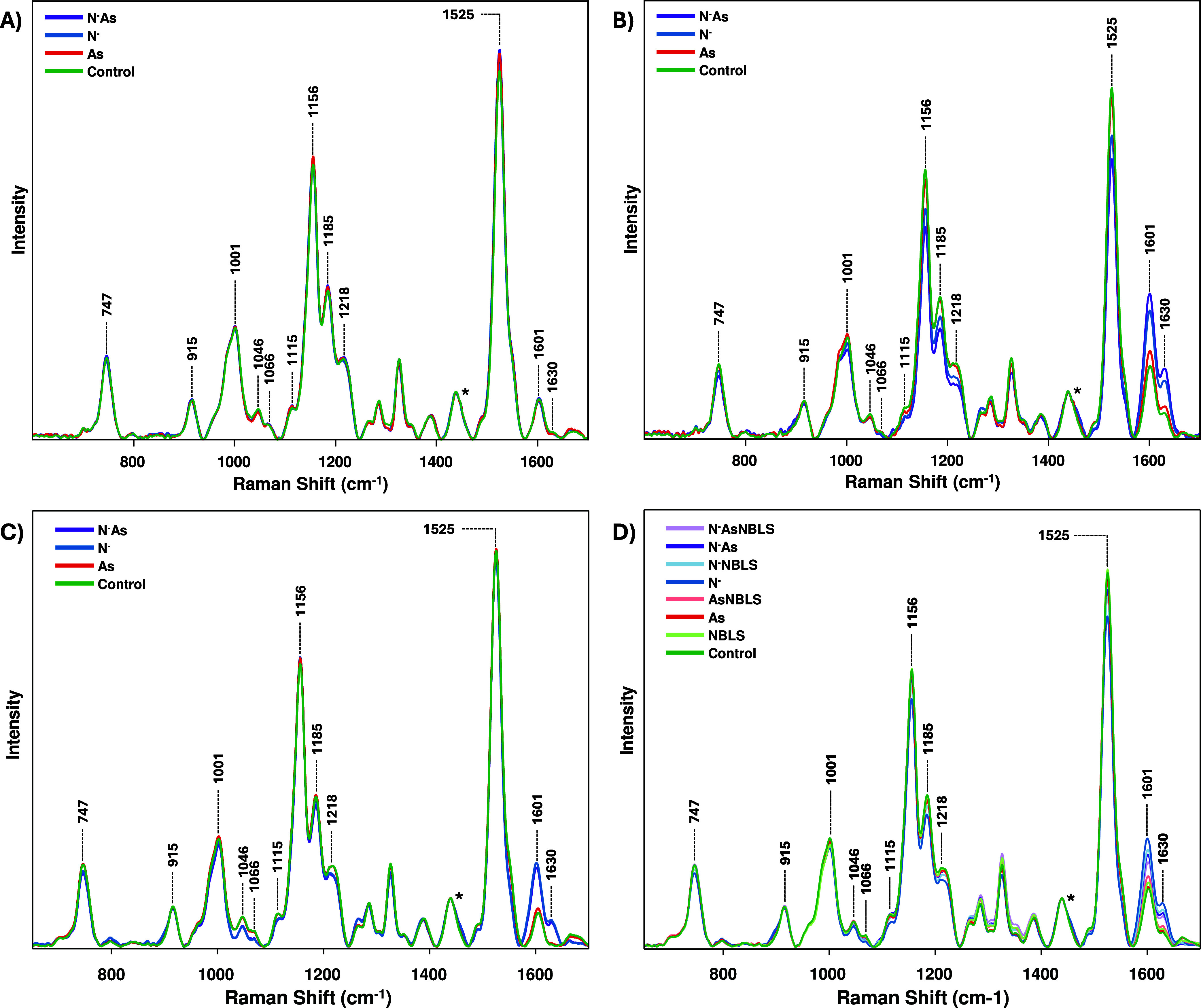
Raman spectra collected from rice leaves of the experimental groups at (A) Experiment 1, week 1, (B) Experiment 1, week 10, (C) Experiment 2, week 5, and (D) Experiment 2, week 10. Asterisk indicates the 1440 cm^–1^ peak, used for normalization.

**Table 1:** Vibrational Bands and Their Assignments for the Raman Spectra Collected from Plant Leaves

band (cm^–1^)	vibrational mode	assignment
480	C–C–O and C–C–C deformations; related to glycosidic ring skeletal deformations δ (C–C–C) + τ(C–O) scissoring of C–C–C and out-of-plane bending of C–O	carbohydrates^53^
520	ν(C–O–C) glycosidic	cellulose^54,55^
747	γ(C–O–H) of COOH	pectin^56^
849–853	(C_6_–C_5_–O_5_–C_1_–O_1_)	pectin^57^
917	ν(C–O–C) in plane, symmetric	cellulose, phenylpropanoids^54^
964–969	δ(CH_2_)	aliphatics^58,59^
1000–1005	in-plane CH_3_ rocking of polyene aromatic ring of phenylalanine	carotenoids;^60^ protein
1048	ν(C–O) + ν(C–C) + δ(C–O–H)	cellulose, phenylpropanoids^54^
1080	ν(C–O) + ν(C–C) + δ(C–O–H)	carbohydrates^53^
1115–1119	sym ν(C–O–C), C–O–H bending	cellulose^54^
1155	C–C stretching; v(C–O–C), v(C–C) in glycosidic linkages, asymmetric ring breathing	carotenoids,^60^ carbohydrates^61^
1185	ν(C–O–H) next to aromatic ring+σ(CH)	carotenoids^60^
1218	δ(C–C–H)	carotenoids,^60^ xylan^62^
1265	guaiacyl ring breathing, C–O stretching (aromatic); –C=C-	phenylpropanoids,^63^ unsaturated fatty acids^64^
1286	δ(C–C–H)	aliphatics^58^
1301	δ(C–C–H) + δ(O–C–H) + δ(C–O–H)	carbohydrates^53,65^
1327	δCH_2_ bending	aliphatics, cellulose, phenylpropanoids^54^
1339	ν(C–O); δ(C–O–H)	carbohydrates^53^
1387	δCH_2_ bending	aliphatics^58^
1443–1446	δ(CH_2_)+ δ(CH_3_)	aliphatics^58^
1515–1535	-C=C- (in plane)	carotenoids^66–68^
1606–1632	ν(C–C) aromatic ring + σ(CH)	phenylpropanoids^69,70^
1654–1660	–C=C–, C=O stretching, amide I	unsaturated fatty acids,^64^ proteins^66^

In the second experiment, at week 5, N^–^ produced significant differences at most peaks compared to both the control and As groups, with lower intensities at amino acid (747, 915 cm^–1^), nitrate (1046 cm^–1^), carotenoid peaks (1156, 1218 cm^–1^), and higher intensities at the phenylpropanoid peaks (1601, 1630 cm^–1^) [Figures 5 and S2]. The N^–^As group showed these same changes, in addition to decreases at the 1001 and 1185 cm^–1^ carotenoid peaks. The N^–^ and N^–^As groups only showed a difference in intensity from each other at the 1185 cm^–1^ peak. In contrast, the As group only exhibited a decrease at the 1156 cm^–1^ and an increase at 1601 cm^–1^ relative to the control. At week 10, 5 weeks postinoculation, all groups except As and AsNBLS differed from the control at the 1001 cm^–1^ carotenoid peak [Figure 5D]. The N^–^ group showed the most pronounced spectral changes, differing significantly at nearly every biologically relevant peak. Furthermore, both the N^–^ and N^–^As groups had significantly reduced nitrate content at the 1046 cm^–1^ peak. This change was notably absent during the co-occurrence of NBLS. All ND groups also displayed elevated intensities at both phenylpropanoid peaks. In general, N^–^ and N^–^NBLS groups exhibited more drastic changes than did N^–^As and N^–^AsNBLS [Figures 6 and S3]. The As group again only exhibited one decrease in peak intensity, now at the 1185 cm^–1^ peak. All abiotic stressors could be differentiated from each other by their intensity at the 1525 cm^–1^ peak. Overall, NBLS did not seem to significantly alter biomolecular content compared to the control, nor did any single biomarker differentiate AsNBLS, N^–^NBLS, and N^–^AsNBLS from their uninoculated counterparts.

**Figure 6:**
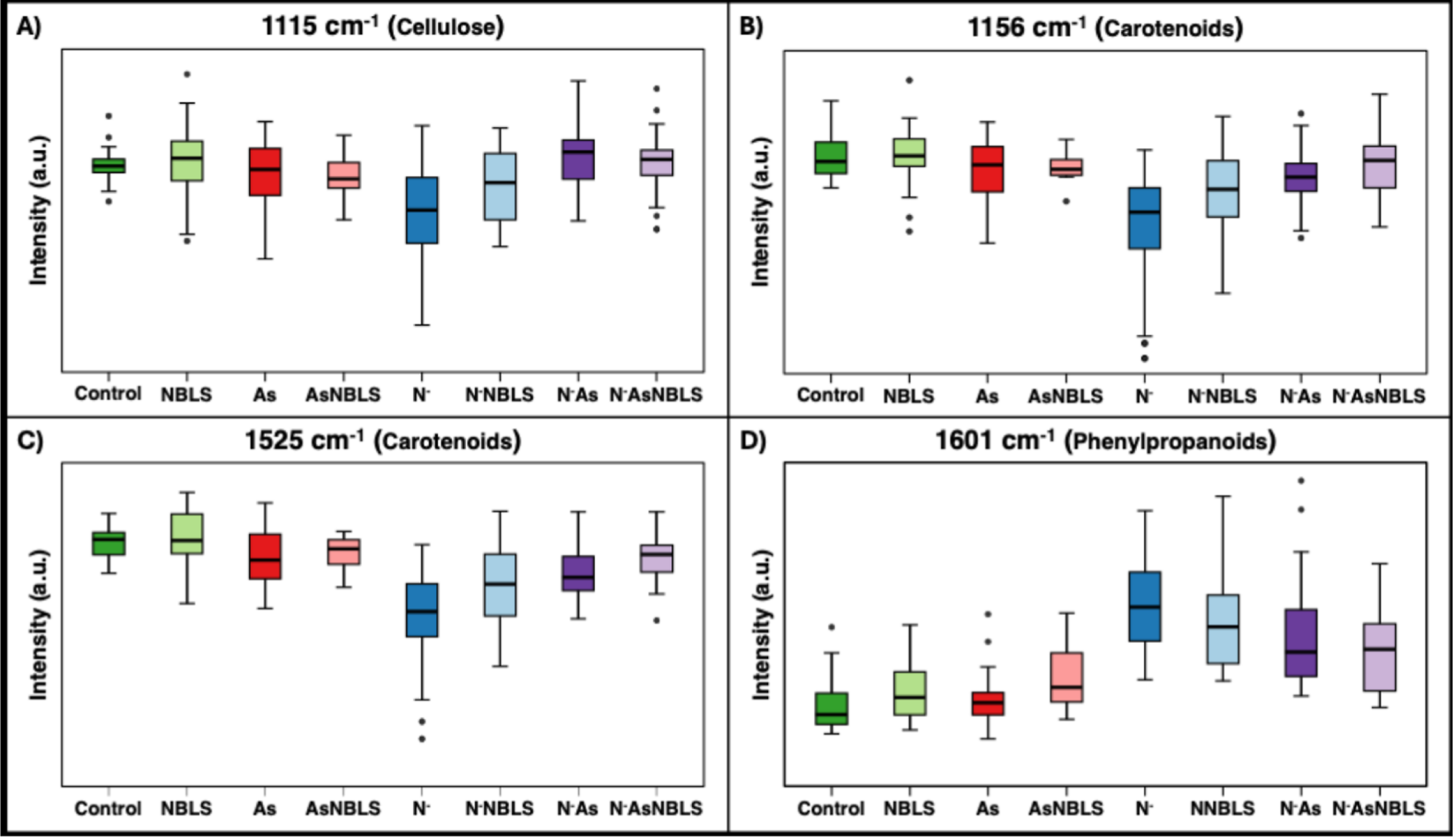
Box-and-whiskers plot of Raman intensity at various biologically relevant peaks from week 10 in Experiment 2 for cellulose (1115 cm^–1^) (A), carotenoids (1156 cm^–1^) (B) and 1525 (C) and phenylpropanoids (1601 cm^–1^) (D).

The temporal peak intensity trends from Experiment 1 revealed several patterns [Figures 7 and S4]. Early after transplantation, spectral intensity trends were similar across experimental groups, except at week 1. After week 6, ND groups sharply diverged from nitrogen-complete groups. A similar trend was observed in the nitrate and lipid peaks, although a statistically significant difference in intensity was lacking at week 10. Arsenic-induced spectral changes only became apparent by week 8 at the phenylpropanoid peaks and by week 9 at the carotenoid and cellulose peaks, although again, a statistical significance was lacking at week 10. Finally, arsenic stress had minimal impact on amino acid peaks, whereas ND led to consistently lower intensities from week 3 onward.

**Figure 7:**
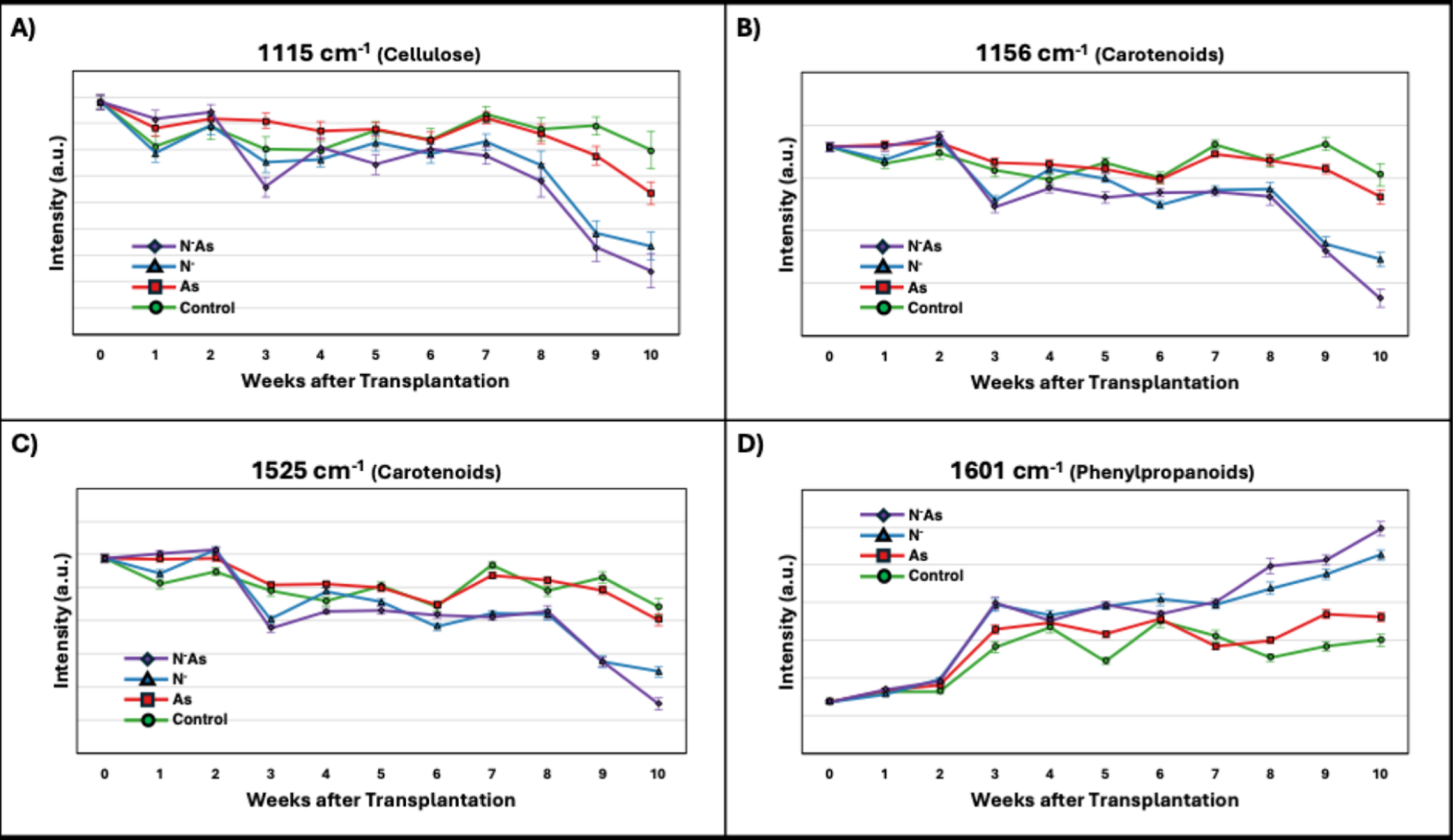
Average Raman intensity of various biologically relevant peaks at each week in Experiment 1. Vertical lines indicate the standard error of the mean for cellulose (1115 cm^–1^) (A), carotenoids (1156 cm^–1^) (B) and 1525 (C) and phenylpropanoids (1601 cm^–1^) (D).

Using 2D-COS with time as the perturbation, we can identify mechanistically linked peak changes and the order in which these changes occur. In the control synchronous spectrum, prominent autopeaks (peaks along the diagonal) at 1525 and 1600 cm^–1^ indicated changes in intensity occur at these peaks as part of normal crop development [Figure 8]. These autopeaks are present in all stress conditions, but ND had an additional autopeak at 1156 cm^–1^, indicating a strong response at this peak to nitrogen deficiency.

**Figure 8:**
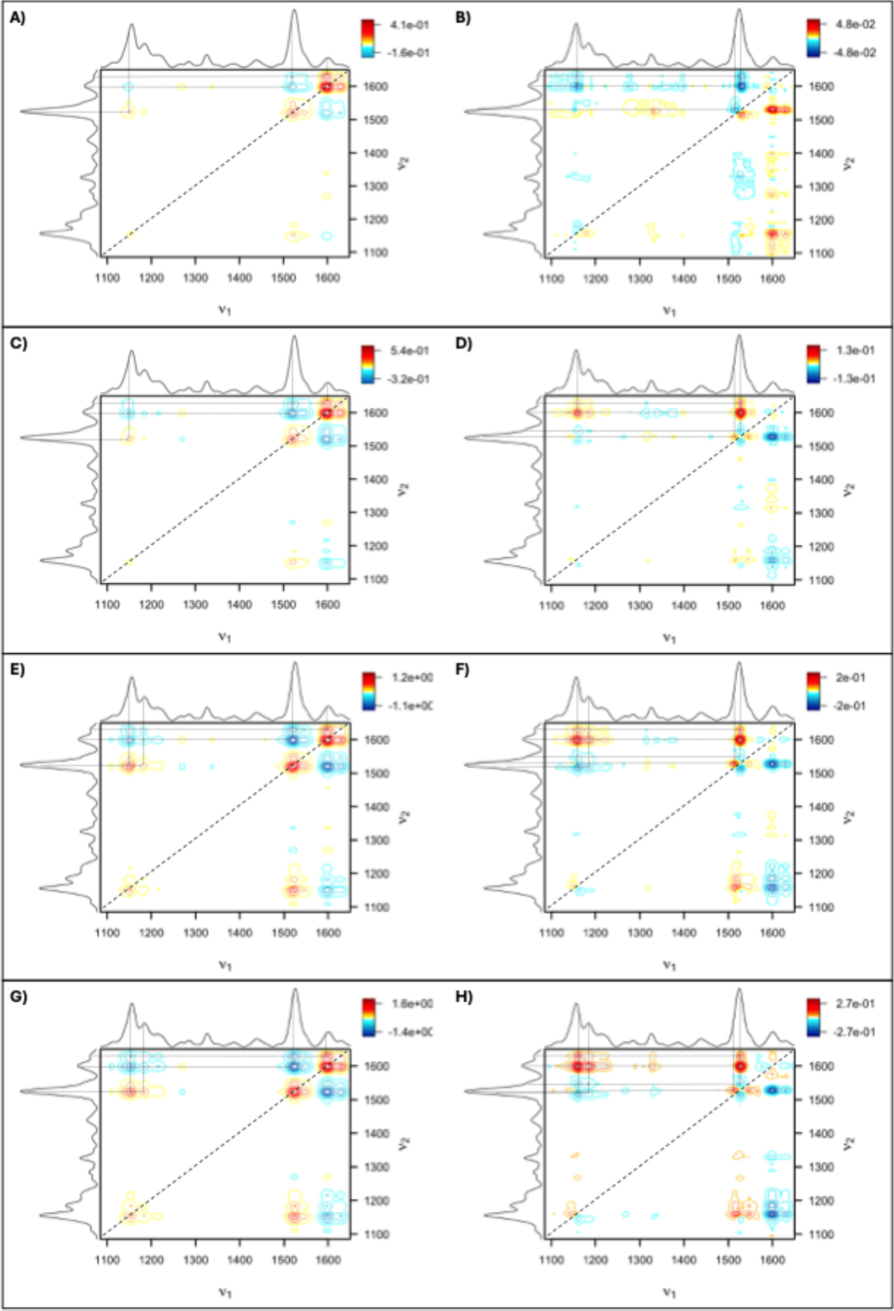
2D-COS analysis of the relationship between stress and time in Experiment 1. Plots (A,C,E,G) are the synchronous spectra for the control, As, N^–^, and N^–^As, respectively. Plots (B,D,F,H) are the asynchronous spectra for the control, As, N^–^, and N^–^As, respectively.

The synchronous spectra of all experimental groups show 2–3 prominent positive cross-peaks (at 1156–1525 cm^–1^, 1601–1630 cm^–1^, and in ND groups, also one at 1185–1525 cm^–1^). This indicated that changes occurring in these peaks occurred in the same direction and could be mechanistically linked. All synchronous spectra contained 3–5 prominent negative cross-peaks (at 1155–1601 cm^–1^, 1525–1601 cm^–1^, 1525–1630 cm^–1^, and in ND groups also at 1155–1630 cm^–1^ and 1185–1600 cm^–1^). This again implied the changes at these peaks were mechanistically linked but that they instead occurred in opposing directions. Overall, spectral changes increased in magnitude from control to the As group, to the N^–^ group, and then to the N^–^As group.

In the control asynchronous spectrum, the cross-peaks at 1155–1525 cm^–1^ and 1155–1601 cm^–1^ indicated that the 1601 cm^–1^ phenylpropanoid peak changes before the carotenoid peaks. Further, peak splitting in the 1525 cm^–1^ region revealed that the 1516 cm^–1^ component shifts after the composite 1525 cm^–1^ peak in healthy rice. In the stressed crops’ asynchronous spectra, this trend reversed. Carotenoid peaks changed before the 1601 and 1630 cm^–1^ phenylpropanoid peaks, and peak splitting at the 1525 cm^–1^ peak was more pronounced, with the 1516 cm^–1^ component peak shifting earlier. A negative cross-peak at 1536–1525 cm^–1^ and 1550–1525 cm^–1^ further supported a sequence in which the 1525 cm^–1^ composite peak shifted last. Lastly, solely in ND groups, additional cross-peaks at 1155–1516 cm^–1^ and 1185–1516 cm^–1^ indicated that the 1516 cm^–1^ component changed before these particular carotenoid peaks.

Peak fitting revealed that component peaks contributing to the 1525 cm^–1^ composite peak existed at 1490, 1516, 1525, 1536, and 1550 cm^–1^ [Figure S5]. The 1516, 1525, and 1536 cm^–1^ peaks correspond to different carotenoid species, with structural variations surrounding the polyene backbone causing red- or blue-shifting. The 1490 and 1550 cm^–1^ shoulders remain poorly characterized but have been linked to DNA/triterpenoids and lipids, respectively.^34–36^ To fit these components, spectra for each experimental condition were averaged into three spectra, and their resultant average area under the curve (AUC) was compared using ANOVA [Table 2]. Due to the collection of only three time points, plotting of temporal trends, 2D-COS analysis, and peak-fitting were not performed for Experiment 2.

**Table 2:** Average Area under the Curve (AUCs) and Percent Changes of Component Peaks (% Δ) after Deconvolution of the 1525 cm^–1^ Composite Peak in Experiment 1 Acquired from Plants at Week 1 (W1) and Week 10 (W10)^a^

Raman shift, cm^–1^	Control			As			N			NAs		
	W1	W10	% Δ	W1	W10	% Δ						
1490	5.71	4.34	–24.0	6.28	4.65	–25.9*	7.08	2.68	–62.1*	5.85	2.36	–59.6***
1516	51.22	45.01	–12.1	58.04	45/97	–20.8*	69.64	32.01	–54.0*	54.74	23.87	–56.4***
1525	121.95	113.31	–7.1	123.41	108.83	–11.8*	103.40	105.17	1.7	132.83	104.31	–21.5**
1536	23.67	24.34	2.8	23.54	21.90	–7.0*	38.77	17.53	–54.8	17.72	13.44	–24.1
1550	26.05	19.22	–26.2	28.39	18.16	–36.0*	18.86	12.46	–33.9	31.34	10.34	–67.0***

a **P < 0.01, ***P < 0.001.

Binary PLS-DA was performed at each week in Experiment 1 to evaluate the reliability of RS in detecting arsenic stress and distinguishing it from nitrogen deficiency. Three sets of PLS-DA models were constructed to evaluate RS’s diagnostic selectivity for arsenic stress and ND, as well as overall diagnostic sensitivity [Table 3, Figures 9 and S6]. The loadings plot for sensitivity at week 10 showed that LV1 primarily captured arsenic stress patterns (60.71% variance), while LV2 captured ND patterns [Figure S7]. Furthermore, arsenic stress was associated with distinct shapes at 1000 and 1525 cm^–1^, particularly the 1490 and 1550 cm^–1^ shoulders. On the other hand, ND correlated with less defined carotenoid and aliphatic peaks and higher amino acid peak intensities. For Experiment 2, binary PLS-DA was performed at week 10 to reassess the reliability of RS in detecting the abiotic stresses, now with NBLS as an additional variable [Table 4].

**Table 3:** PLS-DA True Positive Rates (TPR) and Matthew’s Correlation Coefficient (MCC) for Each Treatment and Experimental Timepoint in Experiment 1

binary TPR				MCC		
week	As selectivity	N^–^ selectivity	sensitivity	As selectivity	N^–^ selectivity	sensitivity
1	90.9	73.1	85.3	0.825	0.458	0.707
2	55.9	75.5	77.6	0.118	0.509	0.553
3	64.8	79.3	86.8	0.294	0.583	0.736
4	61.1	77.3	69.7	0.222	0.549	0.393
5	85.3	77.0	85.1	0.708	0.538	0.702
6	70.1	85.1	79.2	0.400	0.702	0.584
7	71.1	89.9	84.0	0.422	0.797	0.681
8	85.2	92.4	91.4	0.708	0.843	0.805
9	72.6	98.8	97.2	0.449	0.974	0.944
10	76.1	93.4	95.2	0.518	0.868	0.903

**Figure 9:**
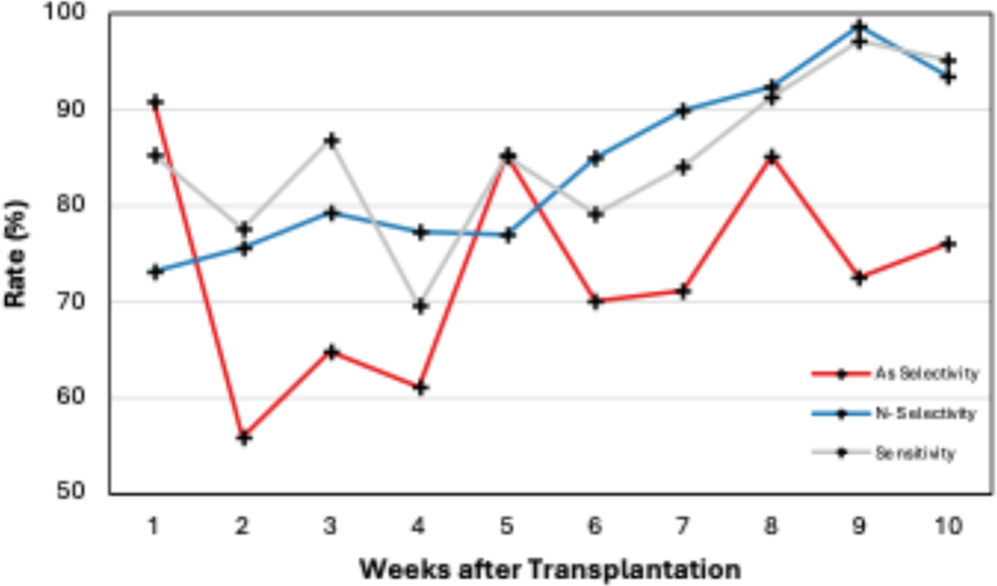
Graph of PLS-DA model selectivity and sensitivity plotted at each time point.

**Table 4:** PLS-DA True Positive Rates (TPR) and Matthew’s Correlation Coefficients (MCC) for Each Treatment at Week 10 in Experiment 2

	week 10	
binary TPR	As selectivity	67.0
	N^–^ selectivity	92.5
	NBLS selectivity	79.4
	sensitivity	97.2
MCC	As selectivity	0.340
	N^–^ selectivity	0.848
	NBLS selectivity	0.588
	sensitivity	0.944

In both experiments, ND induced greater spectral changes over time than arsenic stress, however, in Experiment 1, arsenic effects were most pronounced at week 1. This early response to arsenic likely resulted from the seedlings’ vulnerability during transplantation shock. Transplantation shock is associated with temporary growth inhibition due to root damage, inducing water deficiency.^37^ At week 1, carotenoid content was elevated in arsenic-treated plants, which was atypical of the expected decrease in content. Both water deficiency and arsenic stress increase ROS levels, but the detoxification pathways may be distinct.^38^ These results suggest that transplanting rice into arsenic-laden soil may initially stimulate carotenoid production. Regardless, this effect disappeared by week 2 in Experiment 1, with there being no differences in plant height or spectral intensity.

The overall temporal peak intensity trends also indicated that after recovering from transplantation shock, crops subjected to these lower levels of arsenic exhibited minimal biochemical responses until late in development. Intensity differences between the arsenic-treated and untreated groups occurred in weeks 8 and 9, corresponding to the flowering and grain-filling stages, when crops allocate most of their resources to seed production. Despite this, arsenic stress remained relatively weak, as indicated by the lack of statistically significant differences in both peak intensities and average plant height at week 10 in Experiment 1, and weeks 5 and 10 in Experiment 2. Notably, in Experiment 2, the As group did not show increased phenylpropanoid content at week 10. However, this can likely be explained by the crops’ delayed development, since they had not yet reached the reproductive stage.

ND was initially minimal in Experiment 1, as early growth stages slowly exhausted the soil’s nitrogen reserves. The 1046 cm^–1^ nitrate peak has previously been associated with ND, and the temporal intensity trend showed decreasing nitrate content in the ND plants.^39^ While ND caused significantly lower intensity at this peak in Experiment 2, it was not statistically significant when measured by RS in Experiment 1. The effects of ND were also much more severe in Experiment 2, suggesting the 1046 cm^–1^ peak might have limited utility as an ND biomarker. Alternatively, amino acid peaks were significantly lower in ND rice by week 3 in Experiment 1, correlating with previous literature showing that free amino acid concentration declines as nitrogen supply depletes.^40^ By week 4, ND plants were also approximately 10 cm shorter than nitrogen-complete plants. However, numerous stressors and nutritional deficiencies can stunt growth, so using the amino acid peaks for early detection before symptoms like chlorosis appear could be critical.

When ND and arsenic stress co-occurred, we observed two distinct patterns between Experiments 1 and 2. In Experiment 1, this stress combination led to a higher phenylpropanoid production than either stressor elicited alone. However, in Experiment 2, ND alone produced a stronger response than when it occurred together with arsenic stress. This difference can likely be explained by variation in the number of leaves. N^–^ and N^–^NBLS plants in Experiment 2 had about twice as many surviving leaves as did N^–^As and N^–^AsNBLS plants, meaning nutrients were divided across more tissue. [Figure 4]. In contrast, the few surviving leaves in N^–^As and N^–^AsNBLS likely receive a greater share of nutrients, which could have masked the severity of stress from a biochemical perspective. This is further evident from the development of chlorosis in the N^–^ and N^–^NBLS rice but not in the N^–^As and N^–^AsNBLS rice. Therefore, we expect that in cases of pronounced ND, the co-occurrence of arsenic stress can induce greater leaf loss, thereby reducing the severity of stress detected by RS. At such stunted growth, however, insights provided by RS are likely of minor consequence.

NBLS infection resulted in a minimal amount of necrotic lesions and only had weak spectral differences at individual peaks. Although not statistically significant, all inoculated plants were, on average, shorter compared to their uninoculated counterparts. Furthermore, the spectra of N^–^NBLS and N^–^AsNBLS exhibited less stress than did N^–^ and N^–^As, while NBLS and AsNBLS showed more stress relative to the control and the As groups, respectively. Although rice is susceptible at all developmental stages, susceptibility is highest around booting to panicle emergence.^30,41^ Due to delayed plant development and cooler temperatures, the rice remained within the vegetative phase for the 5 weeks following inoculation, likely suppressing disease progression to an extent. Still, NBLS remained an important confounding variable, which becomes more apparent upon analyzing all three stressors using PLS-DA.

By matching peaks from the spectra in Experiment 1 to their corresponding vibrational assignments in the 2D-COS spectra, we gained mechanistic insights into the structural changes in biomolecules. From the control spectra, we could link a prior phenylpropanoid accumulation to a later carotenoid decline occurring due to natural leaf senescence and chloroplast degradation.^42,43^ The 1525 cm^–1^ autopeak indicates that carotenoid degradation primarily targets their polyene chain conjugation, a key step in their degradation pathway. The earlier increase in phenylpropanoid content likely reflects their role in regulating plant growth, especially flowering and senescence.^44^

In ND groups’ synchronous spectra, the 1185 cm^–1^ autopeak indicated that a further degradation of the carotenoid end-group was also prominent. The 1185–1525 cm^–1^ cross-peak in the asynchronous spectra suggested that this degradation followed cleavage of the polyene structure. In all stress conditions, asynchronous spectra showed that carotenoid depletion preceded phenylpropanoid accumulation, implying that carotenoids act as the first responders to ND and arsenic stress, while phenylpropanoid production must first be upregulated.^45,46^ Both synchronous and asynchronous spectra confirmed that combined arsenic stress and ND induce a stronger response than either stressor alone, under Experiment 1’s experimental conditions.

The analysis of the split peak at 1525 cm^–1^ suggested that certain carotenoids are preferentially degraded between healthy and stressed rice. The 1516 cm^–1^ component peak is associated with β-carotene, while the 1525 cm^–1^ component is associated with lutein and zeaxanthin.^47–49^ These carotenoids participate in the xanthophyll cycle, where β-carotene and violaxanthin convert to zeaxanthin, which quenches excited chlorophyll generated by high light stress.^50–52^ This stress also leads to excess ROS, similar to arsenic exposure. In all stressed groups, asynchronous spectra indicated that β-carotene degradation preceded the depletion of lutein and zeaxanthin. This could indicate simply that β-carotene was preferentially degraded, but could also imply that β-carotene was converted to zeaxanthin to mitigate ROS before overall carotenoid breakdown. In ND groups, the asynchronous cross-peaks at 1516–1155 cm^–1^ and 1516–1185 cm^–1^ indicated that β-carotene was depleted or converted before the disruption of carotenoid end groups occurred. Nevertheless, across all stressed conditions, the 1525 cm^–1^ composite peak was the last to change.

No significant changes were observed in the control AUCs between weeks 1 and 10 in Experiment 1; however, the arsenic-treated groups exhibited significant decreases in all component peaks, with the largest reductions occurring at the shoulders. In the N^–^ group, the greatest decrease occurred at the 1490 cm^–1^ shoulder. The 1525 and 1550 cm^–1^ peaks showed significant declines only under arsenic stress, nearly doubling in the N^–^As group compared to the As group, indicating a greater sensitivity to combined arsenic stress and ND. Note that peak areas at 1490 and 1525 cm^–1^ did not directly correlate with the composite peaks’ intensity changes between weeks 1 and 10, as total intensity depends on the combined contributions of all component peaks, which varied in height and width between experimental conditions.

The 1516 cm^–1^ and 1525 cm^–1^ component peaks’ AUCs revealed that arsenic stress led to a greater loss of β-carotene than lutein, possibly due to simultaneous degradation and conversion of β-carotene to zeaxanthin. At the 1525 cm^–1^ peak, neither the N^–^ group nor the control had a significant change. This could indicate that 2D-COS changes at this wavenumber reflect variations in the overall composite peak rather than changes in the underlying 1525 cm^–1^ component peak. In contrast, changes in arsenic-stressed groups likely stem from alterations in the 1525 cm^–1^ component peak. Increased statistical power in peak fitting may further clarify these trends.

For Experiment 1, the PLS-DA model best predicted arsenic stress shortly after transplantation; however, performance then sharply declined in accuracy, only surpassing 70% again after 5 weeks. Conversely, ND prediction steadily improved over the course of the study. Sensitivity, measured by comparing the spectra of ND and arsenic-treated crops, ranged from 70% to 86% in the first few weeks before gradually improving around week 7 as the stress became more distinct. From the LVA plot, we observed that the key discriminatory peaks in the first three LVs were at 1000, 1185, 1525, 1601, and 1630 cm^–1^, suggesting that subtle alterations in carotenoids and phenylpropanoids content provide the foundation for RS diagnostics of arsenic stress and ND. Although the first two LVs captured around 75% of the variance, there was a lack of clear separation in the LVA plots, indicating that other LVs also provided critical patterns for classification. The PLS-DA model for Experiment 2, built for week 10, had comparable performance to the same time point in Experiment 1, with accuracies of 67% for arsenic stress and 92.5% for ND. While reinforcing our Experiment 1 results, it also indicates that NBLS did not compromise RS’s ability to diagnose abiotic stress. Furthermore, although the spectral differences between the inoculated and uninoculated groups were not statistically significant, the PLS-DA model still demonstrated 79.4% accuracy in diagnosing NBLS infection. This indicates that RS can detect NBLS with minimal to no visual symptom expression, regardless of the co-occurrence of ND, arsenic stress, or both.

## Conclusion

These results demonstrated, across two Experiments, that RS can reliably diagnose ND, arsenic stress, and NBLS infection in rice, including when they co-occurred. We found that ND generally caused stronger biochemical responses over time than did arsenic stress; however, this did not prevent the detection of arsenic uptake. We also discovered that detection of arsenic stress is largely dependent on the plant developmental stage, with the greatest detection accuracy directly following transplantation. In addition, RS could detect NBLS infection with nearly 80% accuracy, even in the absence of severe symptoms or the co-occurrence of abiotic stress. Overall, our results show that RS has strong potential as a diagnostic tool for monitoring rice health under complex abiotic conditions and biotic infections.

## Supplementary Material



## Notes

ANOVA heatmap of spectroscopic changes in peaks acquired from experimental groups at different time points of plant growth and average Raman intensity of various biologically relevant peaks at each week in experiments 1 and 2 are presented in Figures S1 and S2. Box-and-whiskers plot of Raman intensity at various biologically relevant peaks from week 10 in experiment 2 are shown in Figure S3. Average Raman intensity of various biologically relevant peaks at each week in experiment 1. Vertical lines indicate the standard error of the mean are shown in Figure S4. Deconvolution of the 1525 cm^–1^ composite peak (dotted line) at W1 (dark lines) and W10 (light lines) in experiment 1 is presented in Figure S5. LV and loading plots are shown in Figure S6 and S7 (PDF)
