# Supplementary material for: Specificity of Arsenic Stress Detection by Raman Spectroscopy During Co-Occurrences of Nitrogen Deficiency and Narrow Brown Leaf Spot
Source: Anal Chem. 2025 Dec 18;98(1):661–72. doi: 10.1021/acs.analchem.5c05673 (PMC12809705; doi:10.1021/acs.analchem.5c05673)
Supplement: Supplementary file 1 [file ac5c05673_si_001.pdf]

Supporting Information

Specificity of Arsenic Stress Detection by Raman Spectroscopy During Co-Occurrences of Nitrogen Deficiency and Narrow Brown Leaf Spot

*Isaac D. Juárez[1,2], Myles Russwurm[1], Sabin Khanal[3], Sudip Biswas[4], Endang M. Septiningsih[4], Xin-Gen Shane Zhou[3], Dmitry Kurouski[1,2]**

Email: dkurouski@tamu.edu

1. Department of Biochemistry and Biophysics, Texas A&M University, College Station, Texas 77843, United States
2. Interdisciplinary Faculty of Toxicology, Texas A&M University, College Station, Texas 77843, United States
3. Texas A&M AgriLife Research Center, Beaumont, TX 77713
4. Department of Soil and Crop Sciences, Texas A&M University, College Station, Texas 77843, United States

Table of Content:

Figure S1. Experiment 1 ANOVA heatmap of spectroscopic changes in peaks acquired from experimental groups at week 1 and week 10. Note, Con = control, As = arsenic stress, N^- = nitrogen deficiency, N^-As = combined stress.

Figure S2. Experiment 2 ANOVA heatmap of spectroscopic changes in peaks acquired from experimental groups at week 5 and week 10. Con = control, As = arsenic stress, N- = nitrogen deficiency, NBLS = narrow brown leaf spot. All other groups indicate a combination of stressors.

Figure S3. Box-and-whiskers plot of Raman intensity at various biologically relevant peaks from week 10 in experiment 2.

Figure S4. Average Raman intensity of various biologically relevant peaks at each week in experiment 1. Vertical lines indicate the standard error of the mean.

Figure S5. Deconvolution of the 1525 cm^{-1} composite peak (dotted line) at W1 (dark lines) and W10 (light lines) in experiment 1.

Figure S6. Latent variable analysis plots of the trial 1 PLS-DA models for (A) As stress selectivity, (B) N deficiency selectivity, and (C) sensitivity between the two at W10.

Figure S7. Loading plot for the trial 1 PLS-DA sensitivity model at W10, illustrating the contribution of the most important variables for the separation of spectra between nitrogen deficiency (N) and arsenic stress (As).

	747	915	1001	1046	1066	1115	1156	1185	1218	1525	1601	1630
W10 – Kruskall-Wallis	< .001	0.001	< .001	0.108	0.008	< .001	< .001	< .001	< .001	< .001	< .001	< .001
Con – As	0.670	0.277	0.242	NS	0.992	0.067	0.203	0.661	0.348	0.409	0.033	0.105
Con – N⁻	< .001	0.112	0.038	NS	0.095	< .001	< .001	< .001	< .001	< .001	< .001	< .001
Con – N⁻As	< .001	< .001	< .001	NS	0.015	< .001	< .001	< .001	< .001	< .001	< .001	< .001
As – N⁻	< .001	0.527	< .001	NS	0.040	0.004	< .001	< .001	< .001	< .001	< .001	< .001
As – N⁻As	< .001	0.002	< .001	NS	0.003	< .001	< .001	< .001	< .001	< .001	< .001	< .001
N⁻ – N⁻As	0.007	0.015	0.006	NS	0.350	0.194	0.008	0.010	0.011	0.002	0.063	0.036
W1 – Kruskal-Wallis	< .001	0.004	0.707	0.286	0.737	0.007	0.004	< .001	0.003	< .001	0.096	0.249
Con – As	0.498	0.599	NS	NS	NS	0.181	0.006	0.029	0.640	< .001	NS	NS
Con – N⁻	0.007	0.302	NS	NS	NS	0.792	0.511	0.813	0.071	0.209	NS	NS
Con – N⁻As	0.129	0.074	NS	NS	NS	0.018	0.009	0.003	0.277	< .001	NS	NS
As – N⁻	0.014	0.537	NS	NS	NS	0.051	0.012	0.003	0.006	< .001	NS	NS
As – N⁻As	0.007	0.005	NS	NS	NS	0.206	0.874	0.365	0.451	0.378	NS	NS
N⁻ – N⁻As	< .001	< .001	NS	NS	NS	0.001	0.018	< .001	< .001	< .001	NS	NS

NS	≥0.05
*	<0.05
**	<0.01
***	<0.001

Figure S8. Experiment 1 ANOVA heatmap of spectroscopic changes in peaks acquired from experimental groups at week 1 and week 10. Note, Con = control, As = arsenic stress, N^- = nitrogen deficiency, N^-As = combined stress.

	747	915	1001	1046	1066	1115	1156	1185	1218	1525	1601	1630
W10 – Kruskal-Wallis	< .001	0.002	< .001	0.002	< .001	< .001	< .001	< .001	< .001	< .001	< .001	< .001
Con – NBLS	0.192	0.945	< .001	0.917	0.456	0.508	0.694	0.275	0.752	0.76	0.173	0.134
Con – As	0.602	0.984	0.058	0.666	0.389	0.573	0.23	0.034	0.065	0.057	0.582	0.444
Con – AsNBLS	0.763	0.347	0.409	0.778	0.502	0.213	0.28	0.041	0.017	0.309	0.019	0.026
Con – N^-	< .001	0.009	< .001	< .001	< .001	< .001	< .001	< .001	< .001	< .001	< .001	< .001
Con – N^-NBLS	0.004	0.897	< .001	0.169	< .001	0.095	< .001	< .001	< .001	< .001	< .001	< .001
Con – N^-As	0.098	0.631	< .001	0.008	< .001	0.311	0.014	0.069	0.011	< .001	< .001	< .001
Con – N^-AsNBLS	0.416	0.039	0.002	0.342	0.002	0.511	0.349	0.113	0.12	0.057	< .001	< .001
NBLS – As	0.431	0.961	0.042	0.745	0.914	0.222	0.114	0.311	0.131	0.028	0.413	0.459
NBLS – AsNBLS	0.384	0.32	0.007	0.71	0.999	0.067	0.154	0.293	0.038	0.199	0.271	0.388
NBLS – N^-	0.005	0.012	0.478	0.001	< .001	< .001	< .001	< .001	< .001	< .001	< .001	< .001
NBLS – N^-NBLS	0.117	0.952	0.783	0.207	< .001	0.021	< .001	0.004	< .001	< .001	< .001	< .001
NBLS – N^-As	0.727	0.586	0.631	0.012	< .001	0.727	0.005	0.469	0.027	< .001	< .001	< .001
NBLS – N^-AsNBLS	0.036	0.034	0.44	0.402	0.018	0.997	0.187	0.625	0.219	0.028	< .001	< .001
As – AsNBLS	0.869	0.338	0.383	0.504	0.921	0.459	0.994	0.886	0.465	0.492	0.065	0.125
As – N^-	< .001	0.01	0.006	0.003	< .001	0.003	< .001	< .001	< .001	< .001	< .001	< .001
As – N^-NBLS	0.018	0.912	0.021	0.343	< .001	0.268	0.029	0.057	0.022	0.019	< .001	< .001
As – N^-As	0.254	0.617	0.012	0.027	< .001	0.116	0.204	0.776	0.47	0.034	< .001	< .001
As – N^-AsNBLS	0.183	0.037	0.208	0.602	0.023	0.224	0.801	0.602	0.787	0.988	< .001	< .001
AsNBLS – N^-	< .001	0.001	< .001	< .001	< .001	0.056	< .001	0.001	< .001	< .001	< .001	< .001
AsNBLS – N^-NBLS	0.022	0.294	0.003	0.13	< .001	0.796	0.052	0.118	0.183	0.005	< .001	< .001
AsNBLS – N^-As	0.236	0.615	0.002	0.008	0.002	0.032	0.258	0.691	0.938	0.01	0.007	0.005
AsNBLS – N^-AsNBLS	0.304	0.359	0.046	0.258	0.033	0.068	0.815	0.542	0.333	0.486	0.043	0.011
N^- – N^-NBLS	0.203	0.014	0.663	0.047	0.512	0.067	0.015	0.06	0.013	0.01	0.205	0.255
N^- – N^-As	0.013	0.002	0.816	0.454	0.374	< .001	< .001	< .001	< .001	0.005	0.036	0.035
N^- – N^-AsNBLS	< .001	< .001	0.14	0.016	0.043	< .001	< .001	< .001	< .001	< .001	0.005	0.017
N^-NBLS – N^-As	0.223	0.545	0.838	0.212	0.814	0.008	0.366	0.03	0.118	0.822	0.4	0.328
N^-NBLS – N^-AsNBLS	< .001	0.029	0.295	0.672	0.168	0.021	0.016	0.016	0.011	0.021	0.117	0.205
N^-As – N^-AsNBLS	0.014	0.116	0.211	0.095	0.252	0.724	0.131	0.814	0.325	0.037	0.468	0.772

S3

	747	915	1001	1046	1066	1115	1156	1185	1218	1525	1601	1630
W5 – Kruskal-Wallis	< .001	< .001	< .001	< .001	< .001	< .001	0.04	< .001	< .001	0.276	< .001	< .001
Con – As	0.456	0.329	0.075	0.425	0.423	0.648	0.032	0.192	0.55	NS	0.025	0.196
Con – N	< .001	< .001	0.101	< .001	< .001	< .001	0.009	0.107	< .001	NS	< .001	< .001
Con – N-As	< .001	< .001	0.001	< .001	< .001	< .001	0.028	< .001	< .001	NS	< .001	< .001
As – N^-	< .001	0.012	< .001	< .001	< .001	< .001	0.625	0.004	< .001	NS	< .001	< .001
As – N^-As	< .001	< .001	< .001	< .001	< .001	< .001	0.944	< .001	< .001	NS	< .001	< .001
N – N^-As	0.165	0.211	0.113	0.661	0.497	0.893	0.677	0.012	0.38	NS	0.513	0.586

NS	*≥0.05*
*	*<0.05*
**	*<0.01*
***	*<0.001*

Figure S9. Experiment 2 ANOVA heatmap of spectroscopic changes in peaks acquired from experimental groups at week 5 and week 10. Con = control, As = arsenic stress, N- = nitrogen deficiency, NBLS = narrow brown leaf spot. All other groups indicate a combination of stressors.

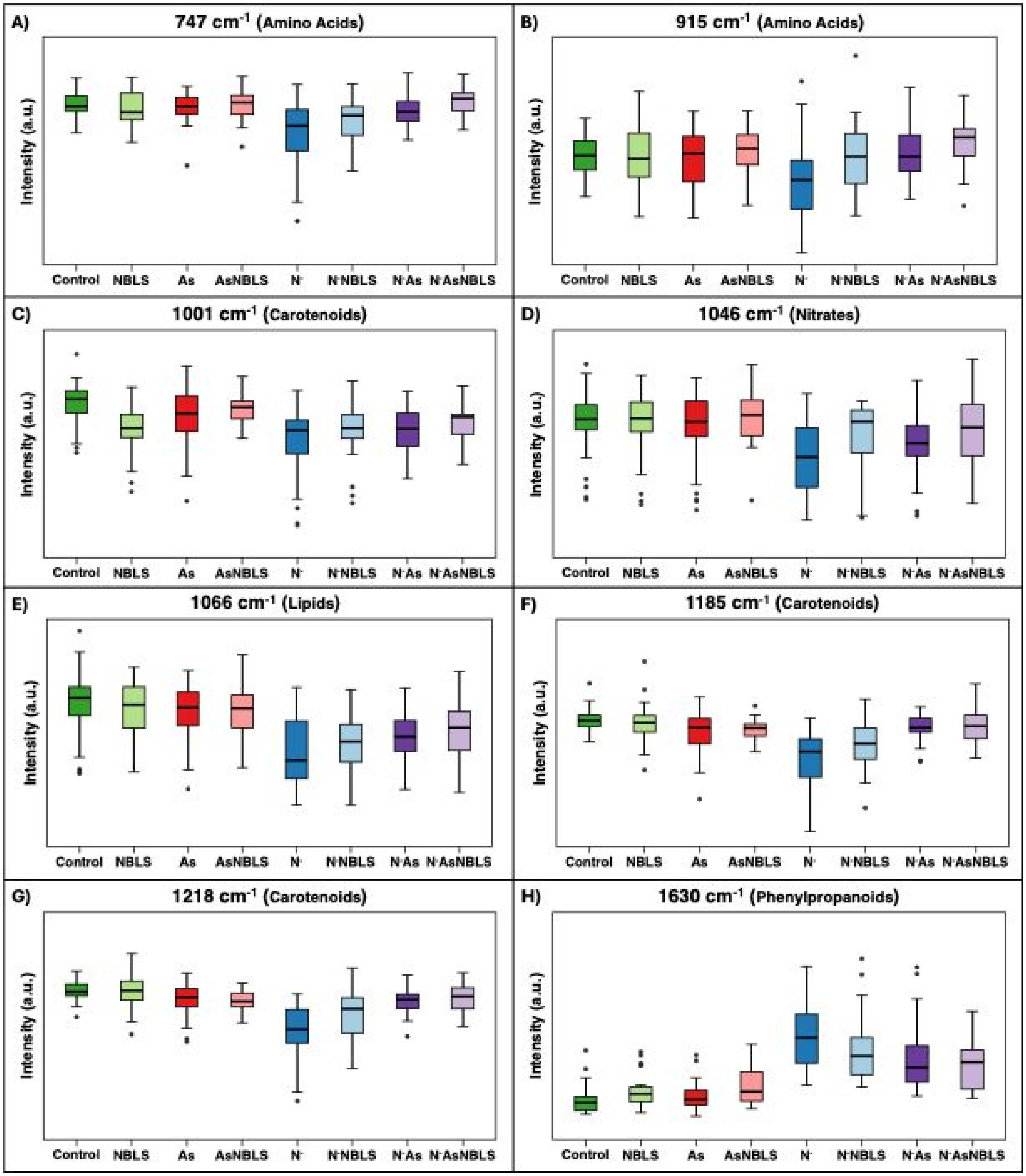

Figure S10. Box-and-whiskers plot of Raman intensity at various biologically relevant peaks from week 10 in experiment 2.

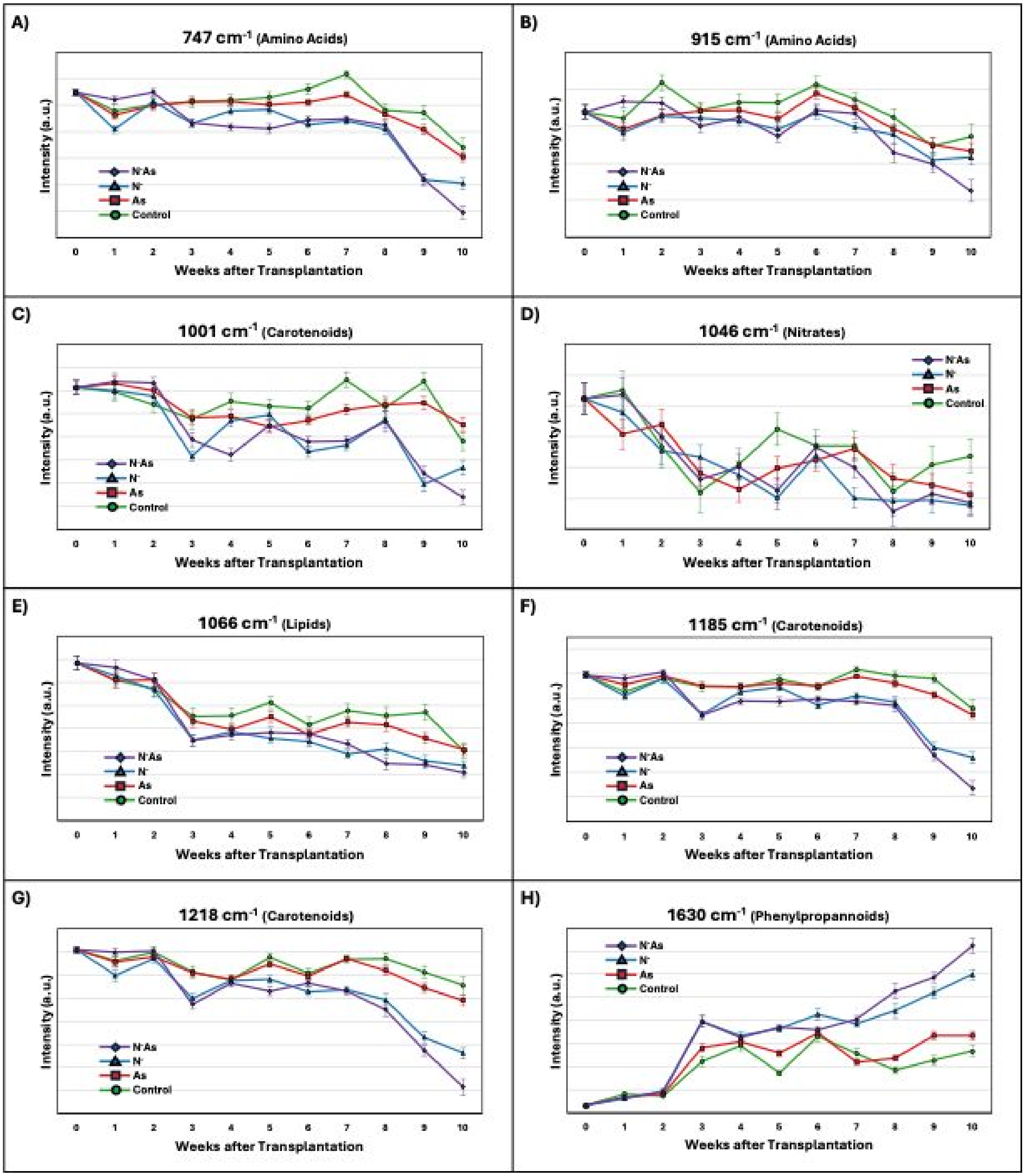

Figure S11. Average Raman intensity of various biologically relevant peaks at each week in experiment 1. Vertical lines indicate the standard error of the mean.

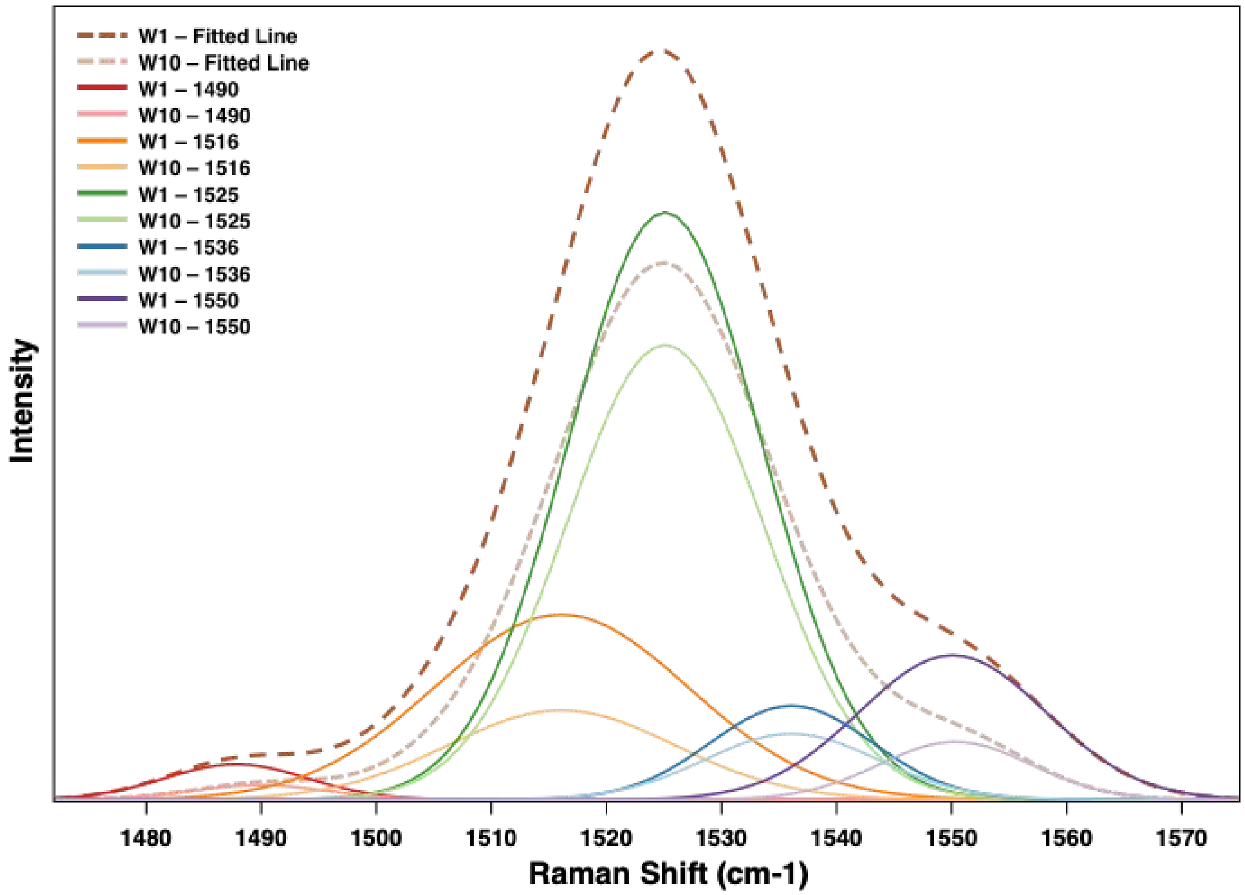

Figure S12. Deconvolution of the 1525 cm^{-1} composite peak (dotted line) at W1 (dark lines) and W10 (light lines) in experiment 1.

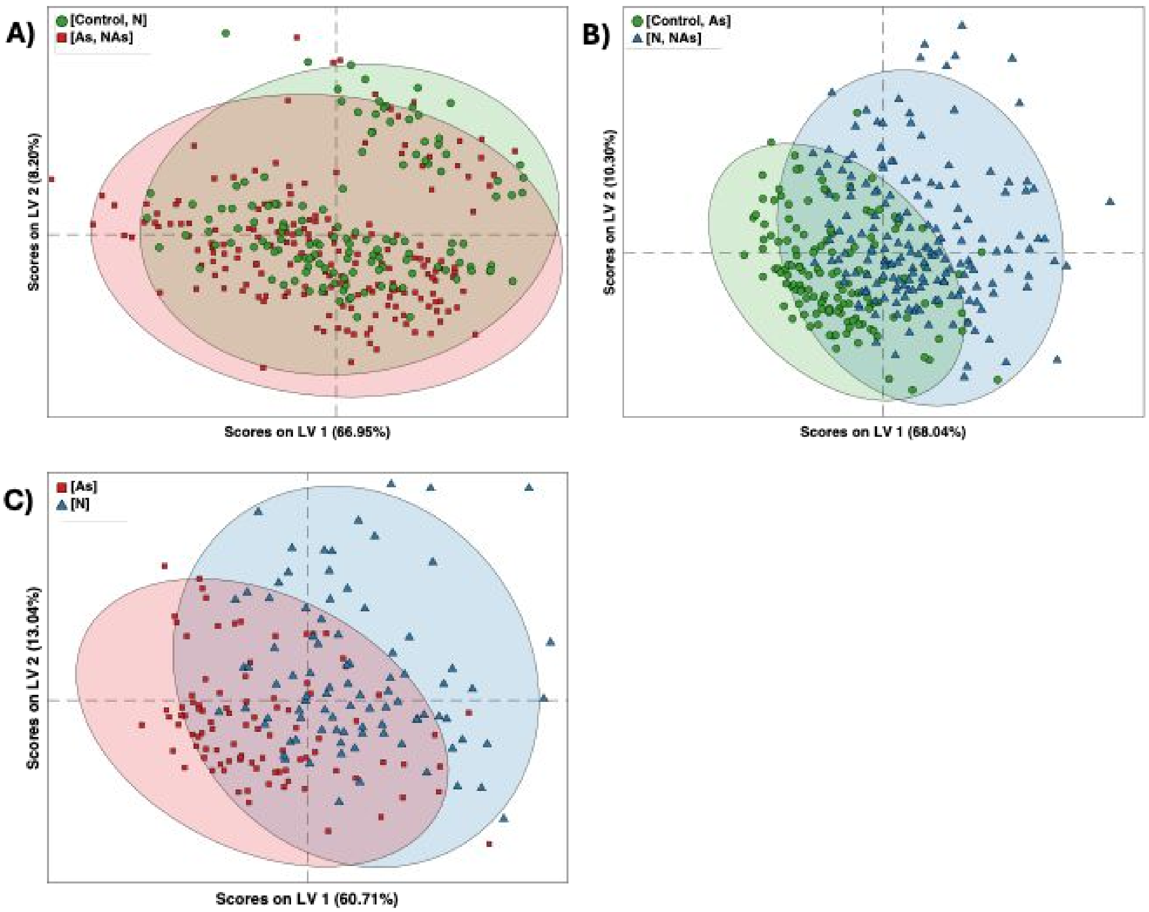

Figure S13. Latent variable analysis plots of the trial 1 PLS-DA models for (A) As stress selectivity, (B) N deficiency selectivity, and (C) sensitivity between the two at W10.

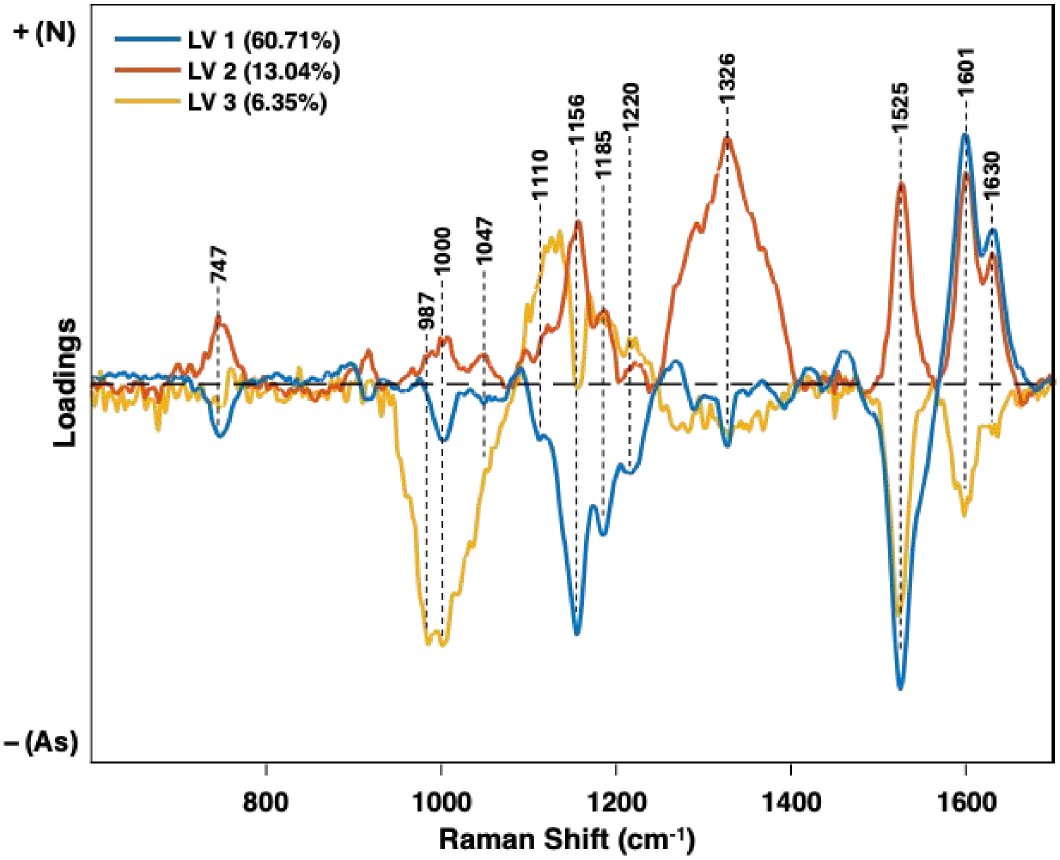

Figure S14. Loading plot for the trial 1 PLS-DA sensitivity model at W10, illustrating the contribution of the most important variables for the separation of spectra between nitrogen deficiency (N) and arsenic stress (As).
